# Supplementary material for: Predicting the uniaxial compressive strength and elasticity modulus of sandstones from physical and mechanical properties using statistical analyses and artificial intelligence-based techniques
Source: PLoS One. 2026 May 15;21(5):e0335703. doi: 10.1371/journal.pone.0335703 (PMC13178931; doi:10.1371/journal.pone.0335703)
Supplement: Attachment — Submitted filename: Revision Notes (R1).docx [file pone.0335703.s002.docx]

**REVISION NOTES**

Manuscript number: PONE-D-25-54958

Title:
 “Predicting the uniaxial compressive strength and elasticity modulus of sandstones from physical and mechanical properties using statistical analyses and artificial intelligence-based techniques”

December 23, 2025

Dear Editor,

I would like to sincerely thank you and the reviewers for your valuable and constructive comments and suggestions on our manuscript. After carefully reviewing all the feedback, we have revised the manuscript accordingly and have tried to address all recommendations as thoroughly as possible. The detailed responses to each of the reviewers’ comments are listed below, point by point. The changes made to the manuscript are highlighted in red in the file titled "Manuscript - Marked-up Version (R1)." It should be noted that all the journal requirements were considered and applied in the text of the manuscript and during its online submission to the journal. This work is based upon research funded by Iran National Science Foundation (INSF) under project No. 4040158. The funder had no role in study design, data collection and analysis, decision to publish, or preparation of the manuscript. We respectfully submit the revised version of the manuscript for your kind consideration and would be grateful if you would evaluate the possibility of its publication. Should you require any further clarifications or additional information, please do not hesitate to contact me. I look forward to hearing from you soon.

Sincerely,

Corresponding author

Journal Requirements:

When submitting your revision, we need you to address these additional requirements.

1. Please ensure that your manuscript meets PLOS ONE's style requirements, including those for file naming. The PLOS ONE style templates can be found at

https://journals.plos.org/plosone/s/file?id=wjVg/PLOSOne_formatting_sample_main_body.pdf and

<https://journals.plos.org/plosone/s/file?id=ba62/PLOSOne_formatting_sample_title_authors_affiliations.pdf>

**Reply;** The comment was considered.

2. In your Methods section, please provide additional information regarding the permits you obtained for the work. Please ensure you have included the full name of the authority that approved the field site access and, if no permits were required, a brief statement explaining why.

**Reply;** The comment was considered. The text was modified based on this comment. Please see the first paragraph of the “Research steps and materials”.

3. Please note that funding information should not appear in any section or other areas of your manuscript. We will only publish funding information present in the Funding Statement section of the online submission form. Please remove any funding-related text from the manuscript.

**Reply;** The comment was considered. The “Declarations” part was removed from the text.

4. Thank you for stating the following financial disclosure:

“This work is based upon research funded by Iran National Science Foundation (INSF) under project No. 4040158.”

Please state what role the funders took in the study.  If the funders had no role, please state: "The funders had no role in study design, data collection and analysis, decision to publish, or preparation of the manuscript."

If this statement is not correct you must amend it as needed.

Please include this amended Role of Funder statement in your cover letter; we will change the online submission form on your behalf.

**Reply;** The comment was considered. The required statements were mentioned in the cover letter.

5. Thank you for stating the following in your Competing Interests section:

“This work is based upon research funded by Iran National Science Foundation (INSF) under project No. 4040158.”

Please complete your Competing Interests on the online submission form to state any Competing Interests. If you have no competing interests, please state "The authors have declared that no competing interests exist.", as detailed online in our guide for authors at http://journals.plos.org/plosone/s/submit-now

 This information should be included in your cover letter; we will change the online submission form on your behalf.

**Reply;** The comment was considered.

6. In the online submission form you indicate that your data is not available for proprietary reasons and have provided a contact point for accessing this data. Please note that your current contact point is a co-author on this manuscript. According to our Data Policy, the contact point must not be an author on the manuscript and must be an institutional contact, ideally not an individual. Please revise your data statement to a non-author institutional point of contact, such as a data access or ethics committee, and send this to us via return email. Please also include contact information for the third party organization, and please include the full citation of where the data can be found.

**Reply;** The comment was considered.

7. Please include your full ethics statement in the ‘Methods’ section of your manuscript file. In your statement, please include the full name of the IRB or ethics committee who approved or waived your study, as well as whether or not you obtained informed written or verbal consent. If consent was waived for your study, please include this information in your statement as well.

**Reply;** The comment was considered.

8. We note that Figure 2 in your submission contain map/satellite images which may be copyrighted. All PLOS content is published under the Creative Commons Attribution License (CC BY 4.0), which means that the manuscript, images, and Supporting Information files will be freely available online, and any third party is permitted to access, download, copy, distribute, and use these materials in any way, even commercially, with proper attribution. For these reasons, we cannot publish previously copyrighted maps or satellite images created using proprietary data, such as Google software (Google Maps, Street View, and Earth). For more information, see our copyright guidelines:

http://journals.plos.org/plosone/s/licenses-and-copyright.

**Reply:** The authors of the manuscript have drawn a general map of Iran by themselves and provided required information on the map that it doesn’t need to any license or copyright permission.

We require you to either (1) present written permission from the copyright holder to publish these figures specifically under the CC BY 4.0 license, or (2) remove the figures from your submission:

 1. You may seek permission from the original copyright holder of Figure 2 to publish the content specifically under the CC BY 4.0 license.

 We recommend that you contact the original copyright holder with the Content Permission Form (http://journals.plos.org/plosone/s/file?id=7c09/content-permission-form.pdf) and the following text:

“I request permission for the open-access journal PLOS ONE to publish XXX under the Creative Commons Attribution License (CCAL) CC BY 4.0 (http://creativecommons.org/licenses/by/4.0/). Please be aware that this license allows unrestricted use and distribution, even commercially, by third parties. Please reply and provide explicit written permission to publish XXX under a CC BY license and complete the attached form.”

 Please upload the completed Content Permission Form or other proof of granted permissions as an "Other" file with your submission.

 In the figure caption of the copyrighted figure, please include the following text: “Reprinted from [ref] under a CC BY license, with permission from [name of publisher], original copyright [original copyright year].”

 2. If you are unable to obtain permission from the original copyright holder to publish these figures under the CC BY 4.0 license or if the copyright holder’s requirements are incompatible with the CC BY 4.0 license, please either i) remove the figure or ii) supply a replacement figure that complies with the CC BY 4.0 license. Please check copyright information on all replacement figures and update the figure caption with source information. If applicable, please specify in the figure caption text when a figure is similar but not identical to the original image and is therefore for illustrative purposes only.

The following resources for replacing copyrighted map figures may be helpful:

 USGS National Map Viewer (public domain): http://viewer.nationalmap.gov/viewer/

The Gateway to Astronaut Photography of Earth (public domain): http://eol.jsc.nasa.gov/sseop/clickmap/

Maps at the CIA (public domain): https://www.cia.gov/library/publications/the-world-factbook/index.html and https://www.cia.gov/library/publications/cia-maps-publications/index.html

NASA Earth Observatory (public domain): http://earthobservatory.nasa.gov/

Landsat: http://landsat.visibleearth.nasa.gov/

USGS EROS (Earth Resources Observatory and Science (EROS) Center) (public domain): http://eros.usgs.gov/#

Natural Earth (public domain): http://www.naturalearthdata.com/

9. If the reviewer comments include a recommendation to cite specific previously published works, please review and evaluate these publications to determine whether they are relevant and should be cited. There is no requirement to cite these works unless the editor has indicated otherwise.

**Reply;** The comment was considered.

Response to the comments of Reviewer #1

We appreciate the reviewer’s constructive feedback. We have substantially revised the manuscript to (1) clarify the analytical comparisons among classification systems, (2) strengthen the methodological justification and parameter derivations, (3) improve figures, tables, and writing clarity, and (4) highlight the broader scientific and international relevance of the study.

Reviewer #1: My comments on the manuscript titled "Predicting the uniaxial compressive strength and elasticity modulus of sandstones from physical and mechanical properties using statistical analyses and artificial intelligence-based techniques" are below;

- The novelty of the article is not stated in the Introduction. Numerous studies exist on predicting the UCS and elasticity of different rock types based on the properties of other rocks. Furthermore, many studies have used artificial intelligence. Therefore, the authors should mention the novelty of their work.

**Reply;** The comment is accepted. We added a new descriptive paragraph for providing the novelty of the work to the Introduction part of the manuscript as: … “The novelty of this study lies in its integrated and systematic framework that combines comprehensive statistical analyses (covariance determination, multiple correlation metrics, and simple regression) with a comparative evaluation of multiple artificial intelligence techniques (ANN, kNN, and RF) specifically applied to sandstones from four distinct Iranian geological formations. Unlike many previous studies that focus on either statistical approaches or a single AI method, this research simultaneously evaluates traditional and AI-based models using an identical dataset, allowing for a transparent performance comparison and robustness assessment. In addition, the study emphasizes the use of easily obtainable, non-destructive physical and mechanical parameters and provides detailed sensitivity analysis to quantify the relative importance of input variables, identifying Schmidt hammer rebound as the most influential predictor for both UCS and elasticity modulus. The formation-based interpretation of results further distinguishes this work by linking predictive performance to petro-physical variability among sandstones, thereby enhancing the practical applicability of the proposed models for site-specific geotechnical design where direct laboratory testing is limited.”

- The authors noted that the samples were washed and dried before laboratory testing. It should be noted that this could alter the mechanical properties of the samples, particularly UCS. A statement regarding this should be added to the relevant section.

**Reply;** We appreciate this insightful comment. In this study, washing and oven-drying the specimens at 105 °C were performed solely to remove surface dust, cutting residues, and free moisture, in accordance with ISRM (2007) recommendations, and to ensure consistent initial conditions for physical property measurements. This procedure is widely adopted in rock mechanics studies and is not expected to significantly alter the intrinsic mechanical behavior of intact sandstones, particularly for dense, low- to moderately porous materials such as those investigated here. Also, these specimens experienced moisture and water absorption once during coring with a coring machine and cutting off their heads and bottoms, and the washing operation was performed after cutting off the heads and bottoms of the specimens with a cutter.

So, all specimens were subjected to the specimen preparation protocol, ensuring that any potential minor influence of drying on UCS and elasticity modulus would be systematic and would not affect the comparative analysis, correlations, or predictive modeling results.

- The authors mentioned that "The petrographic investigations were done by optical microscopy .."in Section 2.2. However, no results were given regarding the mineralogical properties of the rocks, and they were not used in the manuscript.

**Reply;** We thank the reviewer for this important observation. In this study, the petrographic investigations were conducted to ensure proper lithological identification, and to identify correct lithological name of the studied sandstones. The related text was corrected based on this comment.

- Table 1 shows that the physical and mechanical properties of samples selected from the same region vary within a narrow range. The implications of this for statistical analyses should be discussed. Furthermore, the suitability of the resulting equations for general use should also be evaluated.

**Reply;** We appreciate this constructive comment. The relatively narrow range of physical and mechanical properties observed within samples from the same formation reflects their similar depositional environment, mineralogical composition, and diagenetic history, which is typical for sandstones originating from a single geological unit. From a statistical perspective, this homogeneity enhances internal consistency and reduces noise, allowing more reliable identification of intrinsic relationships between input parameters and UCS and elasticity modulus, as evidenced by the high correlation coefficients and low prediction errors. However, it is acknowledged that limited intra-formation variability may constrain the extrapolation of the derived regression equations beyond the studied property ranges. Accordingly, the developed equations and AI models are most suitable for sandstones with comparable lithological characteristics and physical property ranges, and their direct application to other rock types or highly heterogeneous sandstones should be undertaken with caution. This limitation was discussed in the manuscript.

- It is already known that there are significant relationships between UCS values of rocks and, in particular, point load and Schmidt hardness values. Indeed, models have been developed to estimate UCS from point load and Schmidt hardness values. Therefore, it is expected that the models obtained in this study would also have high correlation values. This limits the originality of the study.

**Reply;** We acknowledge the reviewer’s point and agree that strong correlations between UCS, point load index, and Schmidt rebound hardness are well established in the literature. In my opinion, the correlations between UCS, point load index, and Schmidt rebound hardness are not very bad based on the obtained coefficient of determination (R^2^) and other indicators (please see Tables 8, 9, 10, 11 and 12). However, the originality of this study does not stem from the mere existence of these correlations, but from the way they are systematically quantified, compared, and integrated within a unified analytical framework. Unlike many previous studies that focus on developing a single empirical equation, this research concurrently evaluates classical statistical models and multiple AI-based techniques using the same dataset, allowing a transparent assessment of their relative predictive performance. In addition, the study extends beyond UCS by simultaneously modeling the elasticity modulus, incorporates formation-based interpretations, and applies sensitivity analysis to objectively rank predictor importance rather than assuming it a priori. The high correlation values observed therefore serve as validation of data quality and model robustness rather than a redundancy, demonstrating that AI-based approaches can significantly improve prediction accuracy and reliability over conventional regression models, particularly for site-specific sandstone characterization.

- The differences between the artificial intelligence-supported models used should be given in detail.

**Reply;** Thank you for this comment. The artificial intelligence–supported models employed in this study differ fundamentally in their learning mechanisms, data handling strategies, and prediction behavior, which justifies their comparative use. The Artificial Neural Network (ANN) is a parametric, nonlinear model capable of capturing complex interactions among input variables through hidden-layer neurons and weighted connections, making it particularly effective for modeling highly nonlinear relationships between physical–mechanical properties and UCS/E. In contrast, the K-Nearest Neighbors (kNN) algorithm is a non-parametric, instance-based learner that predicts outputs based on similarity measures in the feature space, making it sensitive to data distribution and scaling but advantageous for small datasets without requiring explicit training. The Random Forest (RF) model is an ensemble, tree-based method that combines multiple decision trees using bootstrap aggregation, improving generalization performance and reducing overfitting while providing insights into variable importance. By applying these conceptually distinct AI techniques to the same dataset, this study highlights their respective strengths and limitations in predicting sandstone UCS and elasticity modulus, with ANN demonstrating superior accuracy, kNN offering simplicity and transparency, and RF providing robustness and interpretability.

- Some explanations such as "The demonstrated effectiveness of non-destructive methods, particularly Schmidt hammer and point load tests, provides viable alternatives to conventional laboratory testing, especially in challenging field conditions." given in the Conclusion section are repetitive of results found in many previous studies. Therefore, it is understood that the study does not contain original results.

**Reply;** We appreciate the reviewer’s concern and acknowledge that the effectiveness of Schmidt hammer and point load tests as indirect indicators of UCS has been widely reported in previous studies. However, in this study, such statements are not presented as novel discoveries but rather as outcome-based confirmations derived from a comprehensive, formation-specific dataset and a comparative modeling framework. The originality of the results lies in demonstrating how these non-destructive parameters perform within an integrated statistical–AI environment, quantifying their relative influence through sensitivity analysis, and verifying their predictive power simultaneously for both UCS and elasticity modulus in sandstones from distinct geological formations. The conclusion emphasizes practical implications by contextualizing well-known methods within a robust, validated modeling strategy rather than claiming methodological novelty. The last paragraph of the Conclusions part was revised based on this comment.

Response to the comments of Reviewer #2

We sincerely thank the reviewer for their careful evaluation and valuable suggestions. We have made extensive revisions to improve methodological clarity, data transparency, and the integration of geological and engineering interpretations. The revised manuscript now includes statistical analysis, clearer terminology, expanded discussion of engineering implications, and improved organization.

Reviewer #2: The following comments need to be addressed for further improvement of paper.

1. This paper explored the application of the AI techniques for the prediction of UCS and Elastic modulus based on the laboratory tests data. The findings of the paper may be helpful the rock engineering community. However, during the review it was seen that the abstract doesn't contain the performance of the used AI models.

**Reply;** Thank you for your thoughtful review and constructive feedback. The abstract indeed focused on the highest-performing AI model (ANN) and key statistical correlations, but did not explicitly compare the performance metrics of all three AI techniques evaluated. The abstract was revised to include a brief comparative summary of the AI model performances based on this comment.

2. The introduction section need to be revised by including the latest literature. In this regard some of the literature is given as: Multiple stress memory characteristic of rocks under uniaxial deflection loading: Insights from acoustic emission signals, Compressive damage constitutive model for brittle coal based on the compaction effect and linear energy dissipation law, Fracture features of brittle coal under uniaxial and cyclic compression loads, Numerical
analysis on the factors affecting post-peak characteristics of coal under uniaxial compression, Localization of Acoustic Emission Source in Rock Using SMIGWO Algorithm, Study on seismic displacement of coal-rock fracture based on radiation energy, Mechanism and research progress of ultrasonic excitation flow enhancement in low permeability coal seam, Slope stability prediction based on a long short-term memory neural network: comparisons with convolutional neural networks, support vector machines and random forest models, Uncertainties of landslide susceptibility prediction: influences of different study area scales and mapping unit scales, Appraisal of Different Artificial Intelligence Techniques for the Prediction of Marble Strength, An Appropriate Model for the Prediction of Rock Mass Deformation Modulus among Various Artificial Intelligence Models, Application of Machine Learning and Multivariate Statistics to Predict Uniaxial Compressive Strength and Static Young’s Modulus Using Physical Properties under Different Thermal Conditions.

**Reply;** Thank you for providing the suitable new references. The introduction section was revised by some related the references.

3. Authors are advised to include the correlation matrices analysis of the input variable used for the prediction of UCS.

**Reply;** Thank you for the valuable suggestion. The discussions of correlation matrices (Pearson, Spearman, and Kendall) for the input variables used in predicting UCS are comprehensively presented in the paper. Also, some explanations were added to the text after Table 7.

4. A discussion section need to be included and the findings of the research need to be discussed.

**Reply;** Thank you for this essential recommendation. The structure of this article adopts an integrated approach where the interpretation and discussion of results are presented immediately following their presentation within each analytical section. This format—applied to the statistical analyses (Section 3), artificial intelligence-based analyses (Section 4), and their respective subsections—ensures a direct and coherent linkage between data, findings, and their scientific interpretation. It allows for a stepwise discussion of implications as each set of results is unveiled, providing clarity and context before synthesizing the overall conclusions in Section 5. Therefore, a standalone “Discussion” heading has not been included, as the critical analysis and contextualization of results are embedded throughout the results sections themselves.

5. The conclusion section need to be summarized and the main findings shall be included

**Reply;** We appreciate the reviewer’s suggestion. The conclusion section was summarized focusing on the main findings of the research based on this constructing comments. Please see this section.

Finally, the authors would like to thank for your kindly suggestions through the manuscript.

Best regards

Corresponding Author
